# Brain ageing in schizophrenia: evidence from 26 international cohorts via the ENIGMA Schizophrenia consortium

**DOI:** 10.1038/s41380-022-01897-w

**Published:** 2022-12-09

**Authors:** Constantinos Constantinides, Laura K. M. Han, Clara Alloza, Linda Antonella Antonucci, Celso Arango, Rosa Ayesa-Arriola, Nerisa Banaj, Alessandro Bertolino, Stefan Borgwardt, Jason Bruggemann, Juan Bustillo, Oleg Bykhovski, Vince Calhoun, Vaughan Carr, Stanley Catts, Young-Chul Chung, Benedicto Crespo-Facorro, Covadonga M. Díaz-Caneja, Gary Donohoe, Stefan Du Plessis, Jesse Edmond, Stefan Ehrlich, Robin Emsley, Lisa T. Eyler, Paola Fuentes-Claramonte, Foivos Georgiadis, Melissa Green, Amalia Guerrero-Pedraza, Minji Ha, Tim Hahn, Frans A. Henskens, Laurena Holleran, Stephanie Homan, Philipp Homan, Neda Jahanshad, Joost Janssen, Ellen Ji, Stefan Kaiser, Vasily Kaleda, Minah Kim, Woo-Sung Kim, Matthias Kirschner, Peter Kochunov, Yoo Bin Kwak, Jun Soo Kwon, Irina Lebedeva, Jingyu Liu, Patricia Mitchie, Stijn Michielse, David Mothersill, Bryan Mowry, Víctor Ortiz-García de la Foz, Christos Pantelis, Giulio Pergola, Fabrizio Piras, Edith Pomarol-Clotet, Adrian Preda, Yann Quidé, Paul E. Rasser, Kelly Rootes-Murdy, Raymond Salvador, Marina Sangiuliano, Salvador Sarró, Ulrich Schall, André Schmidt, Rodney J. Scott, Pierluigi Selvaggi, Kang Sim, Antonin Skoch, Gianfranco Spalletta, Filip Spaniel, Sophia I. Thomopoulos, David Tomecek, Alexander S. Tomyshev, Diana Tordesillas-Gutiérrez, Therese van Amelsvoort, Javier Vázquez-Bourgon, Daniela Vecchio, Aristotle Voineskos, Cynthia S. Weickert, Thomas Weickert, Paul M. Thompson, Lianne Schmaal, Theo G. M. van Erp, Jessica Turner, James H. Cole, ENIGMA Schizophrenia Consortium, Rosa Ayesa-Arriola, Stefan Du Plessis, Yoo Bin Kwak, Víctor Ortiz-García de la Foz, Therese van Amelsvoort, Theo G. M. van Erp, Danai Dima, Esther Walton

**Affiliations:** 1 Department of Psychology, University of Bath, Bath, UK; 2 Centre for Youth Mental Health, The University of Melbourne, Melbourne, VIC Australia; 3 Orygen, Parkville, VIC Australia; 4 Department of Psychiatry, Amsterdam University Medical Centers, Vrije Universiteit and GGZ inGeest, Amsterdam Neuroscience, Amsterdam, The Netherlands; 5 Department of Child and Adolescent Psychiatry, Institute of Psychiatry and Mental Health, Hospital General Universitario Gregorio Marañón, IiSGM, CIBERSAM, School of Medicine, Universidad Complutense, Madrid, Spain; 6 Centro de Investigación Biomédica en Red de Salud Mental (CIBERSAM), Instituto de Salud Carlos III, Spain; 7 Department of Translational Biomedicine and Neuroscience, University of Bari Aldo Moro, Bari, Italy; 8 Department of Psychiatry and Psychotherapy, Ludwig-Maximilians Universität—Munich, Munich, Germany; 9 Department of Psychiatry, Marqués de Valdecilla University Hospital, IDIVAL, School of Medicine, University of Cantabria, Santander, Spain; 10 Laboratory of Neuropsychiatry, IRCCS Santa Lucia Foundation, Rome, Italy; 11 Department of Psychiatry (UPK), University of Basel, Basel, Switzerland; 12 Department of Psychiatry, Psychosomatics and Psychotherapy, University of Lübeck, Lübeck, Germany; 13 School of Psychiatry, University of New South Wales, Sydney, NSW Australia; 14 Neuroscience Research Australia, Sydney, NSW Australia; 15 Department of Psychiatry, University of New Mexico, Albuquerque, NM USA; 16 Department of Psychiatry, Psychiatric University Hospital (UPK), University of Basel, Basel, Switzerland; 17 Division of Addiction Medicine, Centre Hospitalier des Quatre Villes, St. Cloud, France; 18 Tri-institutional Center for Translational Research in Neuroimaging and Data Science (TReNDS), Georgia State, Georgia Tech, Emory, Atlanta, GA USA; 19 Department of Psychiatry, Monash University, Clayton, VIC Australia; 20 School of Medicine, University of Queensland, Herston, QLD Australia; 21 Department of Psychiatry, Jeonbuk National University, Medical School, Jeonju, Korea; 22 Department of Psychiatry, Jeonbuk National University Hospital, Jeonju, Korea; 23 Research Institute of Clinical Medicine of Jeonbuk National University-Biomedical Research Institute of Jeonbuk National University Hospital, Jeonju, Korea; 24 Hospital Universitario Virgen del Rocío, IBiS-CSIC, Universidad de Sevilla, Seville, Spain; 25 Centre for Neuroimaging and Cognitive Genomics (NICOG), School of Psychology, National University of Ireland Galway, Galway, Ireland; 26 Department of Psychiatry, Stellenbosch University, Cape Town, South Africa; 27 Stellenbosch University Genomics of Brain Disorders Research Unit, South African Medical Research Council, Cape Town, South Africa; 28 Department of Psychology, Georgia State University, Atlanta, GA USA; 29 Translational Developmental Neuroscience Section, Division of Psychological and Social Medicine and Developmental Neurosciences, Faculty of Medicine, TU Dresden, Germany; 30 Department of Psychiatry, University of California San Diego, San Diego, CA USA; 31 Desert-Pacific Mental Illness Research Education and Clinical Center, VA San Diego Healthcare System, San Diego, CA USA; 32 FIDMAG Germanes Hospitalàries Research Foundation, Barcelona, Catalonia Spain; 33 Department of Psychiatry, Psychotherapy and Psychosomatics, Psychiatric Hospital, University of Zurich, Zurich, Switzerland; 34 Hospital Benito Menni CASM, Sant Boi de Llobregat, Catalonia Spain; 35 Department of Brain and Cognitive Sciences, Seoul National University College of Natural Sciences, Seoul, South Korea; 36 Institute for Translational Psychiatry, University of Münster, Münster, Germany; 37 School of Medicine & Public Health, The University of Newcastle, Newcastle, NSW Australia; 38 Priority Research Centre for Health Behaviour, The University of Newcastle, Newcastle, NSW Australia; 39 Hunter Medical Research Institute, Newcastle, NSW Australia; 40 Psychiatric University Hospital Zurich, Zurich, Switzerland; 41 Department of Experimental Psychopathology and Psychotherapy, University of Zurich, Zurich, Switzerland; 42 Imaging Genetics Center, Stevens Neuroimaging and Informatics Institute, Keck School of Medicine, University of Southern California, Marina del Rey, CA USA; 43 Division of Adult Psychiatry, Department of Psychiatry, Geneva University Hospitals, Geneva, Switzerland; 44 Mental Health Research Center, Moscow, Russia; 45 Department of Psychiatry, Seoul National University College of Medicine, Seoul, South Korea; 46 Department of Neuropsychiatry, Seoul National University Hospital, Seoul, South Korea; 47 McConnell Brain Imaging Centre, Montreal Neurological Institute and Hospital, McGill University, Montreal, QC Canada; 48 Maryland Psychiatric Research Center, Department of Psychiatry, University of Maryland School of Medicine, Baltimore, MD USA; 49 Department of Computer Science, Georgia State University, Atlanta, GA USA; 50 Neuroscience Institute, Georgia State University, Atlanta, GA USA; 51 School of Psychological Sciences, University of Newcastle, Callaghan, NSW Australia; 52 Department of Neurosurgery, School of Mental Health and Neuroscience, EURON, Maastricht University Medical Centre, Maastricht, The Netherlands; 53 Department of Psychology, School of Business, National College of Ireland, Dublin, Ireland; 54 Queensland Brain Institute, The University of Queensland, Brisbane, QLD Australia; 55 The Queensland Centre for Mental Health Research, The University of Queensland, Brisbane, QLD Australia; 56 Melbourne Neuropsychiatry Centre, Department of Psychiatry, The University of Melbourne & Melbourne Health, Carlton South, VIC Australia; 57 Florey Institute of Neuroscience & Mental Health, Parkville, VIC Australia; 58 Department of Psychiatry and Human Behavior, University of California, Irvine, CA USA; 59 School of Psychology, University of New South Wales, Sydney, NSW Australia; 60 Priority Centre for Brain & Mental Health Research, The University of Newcastle, Newcastle, NSW Australia; 61 School of Biomedical Sciences and Pharmacy, University of Newcastle, Newcastle, NSW Australia; 62 Department of Neuroimaging, Institute of Psychiatry, Psychology and Neuroscience, King’s College London, London, UK; 63 West Region, Institute of Mental Health, Singapore, Singapore; 64 Yong Loo Lin School of Medicine, National University of Singapore, Singapore, Singapore; 65 Lee Kong Chian School of Medicine, Nanyang Technological University, Singapore, Singapore; 66 National Institute of Mental Health, Klecany, Czech Republic; 67 MR unit, Department of Diagnostic and Interventional Radiology, Institute for Clinical and Experimental Medicine, Prague, Czech Republic; 68 Department of Psychiatry and Behavioral Sciences, Baylor College of Medicine, Houston, TX USA; 69 Third Faculty of Medicine, Charles University, Prague, Czech Republic; 70 Institute of Computer Science, Czech Academy of Sciences, Prague, Czech Republic; 71 Faculty of Electrical Engineering, Czech Technical University in Prague, Prague, Czech Republic; 72 Department of Radiology, Marqués de Valdecilla University Hospital, Valdecilla Biomedical Research Institute IDIVAL, Santander, Spain; 73 Advanced Computation and e-Science, Instituto de Física de Cantabria CSIC, Santander, Spain; 74 Department of Psychiatry and Neuropsychology, Maastricht University, Maastricht, The Netherlands; 75 Campbell Family Mental Health Research Institute, CAMH, Toronto, Canada; 76 Department of Psychiatry, University of Toronto, Toronto, ON Canada; 77 Department of Neuroscience and Physiology, SUNY Upstate Medical University, Syracuse, NY USA; 78 Clinical Translational Neuroscience Laboratory, Department of Psychiatry and Human Behavior, University of California Irvine, Irvine, CA USA; 79 Center for the Neurobiology of Learning and Memory, University of California, Irvine, CA USA; 80 Centre for Medical Image Computing, Department of Computer Science, University College London, London, UK; 81 Dementia Research Centre, Queen Square, Institute of Neurology, University College London, London, UK; 82 Department of Psychology, School of Arts and Social Sciences, City, University of London, London, UK

**Keywords:** Schizophrenia, Neuroscience

## Abstract

Schizophrenia (SZ) is associated with an increased risk of life-long cognitive impairments, age-related chronic disease, and premature mortality. We investigated evidence for advanced brain ageing in adult SZ patients, and whether this was associated with clinical characteristics in a prospective meta-analytic study conducted by the ENIGMA Schizophrenia Working Group. The study included data from 26 cohorts worldwide, with a total of 2803 SZ patients (mean age 34.2 years; range 18–72 years; 67% male) and 2598 healthy controls (mean age 33.8 years, range 18–73 years, 55% male). Brain-predicted age was individually estimated using a model trained on independent data based on 68 measures of cortical thickness and surface area, 7 subcortical volumes, lateral ventricular volumes and total intracranial volume, all derived from T1-weighted brain magnetic resonance imaging (MRI) scans. Deviations from a healthy brain ageing trajectory were assessed by the difference between brain-predicted age and chronological age (brain-predicted age difference [brain-PAD]). On average, SZ patients showed a higher brain-PAD of +3.55 years (95% CI: 2.91, 4.19; *I*^2^ = 57.53%) compared to controls, after adjusting for age, sex and site (Cohen’s *d* = 0.48). Among SZ patients, brain-PAD was not associated with specific clinical characteristics (age of onset, duration of illness, symptom severity, or antipsychotic use and dose). This large-scale collaborative study suggests advanced structural brain ageing in SZ. Longitudinal studies of SZ and a range of mental and somatic health outcomes will help to further evaluate the clinical implications of increased brain-PAD and its ability to be influenced by interventions.

## Introduction

Schizophrenia (SZ) is associated with an increased risk of premature mortality, with an average decrease in life expectancy of ~15 years [1–3]. This is partially accounted for by suicidal behaviour or accidental deaths, as well as poor somatic health, including cardiovascular and metabolic disease [4–6]. The high prevalence of physical morbidity, long-term cognitive decline, and excess mortality seen in SZ may partly be the result of “accelerated” ageing (i.e., a biological age which “outpaces” chronological age) [7–9]. An increasing number of studies report systemic, age-related biological changes in SZ patients, including elevated levels of oxidative stress, inflammation, and cytotoxicity [10, 11]. There is also evidence for progressive brain changes in gray and white matter structures that may begin around or after illness onset [12–18], which may, in part, reflect deviations from normal brain ageing trajectories.

Although chronological age can be predicted accurately with neuroimaging data using machine learning, discrepancies can occur between brain-predicted age (also known as “brain age”) and chronological age [19]. This can be referred to as brain-predicted age difference (brain-PAD). A brain-PAD larger than zero indicates a brain that appears “older” than the person’s chronological age, whereas a brain-PAD lower than zero reflects a “younger” brain than expected at a given chronological age. Higher brain-PAD scores have been associated with a wide range of health-related lifestyle factors and outcomes, including smoking, higher alcohol intake, obesity (or higher BMI), cognitive impairments, major depression, type 2 diabetes, and early mortality [20–25].

To our knowledge, only a few studies have investigated brain age in adults with SZ using various machine learning algorithms or imaging (gray and/or white matter) measures. A higher brain-PAD was consistently shown in SZ patients relative to healthy individuals, with reported scores varying from +2.6 to 7.8 years across studies [26–31]. Furthermore, a greater brain-PAD was observed in first-episode SZ patients [26], and longitudinal data suggests that this gap widens predominantly during the first years after illness onset [29]. As these prior studies were performed with relatively small to moderate sample sizes (range: 43–341 patients), it is important to examine whether brain age findings in SZ can be generalised through large-scale studies consisting of many independent samples worldwide. Two recent mega-analyses with up to 1110 SZ patients across multiple cohorts found a moderate increase in brain-PAD derived from structural T1-weighted MRI (Cohen’s *d* = 0.51) [32] and diffusion tensor imaging (Cohen’s *d* = 0.29) [33], respectively. Validation of those findings, as well as identifying which clinical characteristics or other factors may underlie advanced brain ageing in SZ, could have diagnostic and prognostic implications for patients.

Here, we set out to investigate brain age in over 5000 individuals from the Schizophrenia Working Group within the Enhancing Neuro-Imaging Genetics through Meta-analysis (ENIGMA) consortium (26 cohorts, 15 countries), covering almost the entire adult lifespan (18–73 years). We employed a recently developed multisite brain ageing algorithm based on FreeSurfer-derived gray matter regions of interest (ROIs) [24] to examine brain-PAD differences between SZ patients and healthy controls in a prospective meta-analysis. We hypothesised significantly higher brain-PAD in SZ patients, compared to controls. In addition, we assessed whether a higher brain-PAD in SZ patients was associated with clinical characteristics, such as age of onset, length of illness, symptom severity, and antipsychotic treatment.

## Methods

### Study samples

Twenty-six cohorts from the ENIGMA SZ working group with cross-sectional data from SZ patients (*N* = 2803) and healthy controls (*N* = 2598) were included in this study (18–73 years of age). Details of demographics, location, clinical characteristics (including methods for data harmonization), and inclusion/exclusion criteria for each cohort may be found in Supplementary Information (Supplementary Tables S1–3, Supplementary Fig. S1, and Supplementary Material). All sites obtained approval from the appropriate local institutional review boards and ethics committees, and all study participants provided written informed consent.

### Image acquisition and pre-processing

Structural T1-weighted brain MRI scans of each participant were acquired at each site. We used standardized protocols for image analysis and feature extraction (*N*_features_ = 153) across multiple cohorts (http://enigma.ini.usc.edu/protocols/imaging-protocols/). FreeSurfer [34] was used to segment and extract volumes bilaterally for 14 subcortical gray matter regions (nucleus accumbens, amygdala, caudate, hippocampus, pallidum, putamen, and thalamus), 2 lateral ventricles, along with 68 regional cortical thickness and 68 regional cortical surface area measures, and total intracranial volume (ICV). Cortical parcellations were based on the Desikan/Kiliani atlas [35]. Segmentations were visually inspected and statistically examined for outliers. Further details of image acquisition parameters, software descriptions, and quality control may be found in Supplementary Table S4 and Supplementary Material.

### Brain age prediction

We used the publicly available ENIGMA brain age model (https://photon-ai.com/enigma_brainage). As described and discussed in Han et al. [24], brain age models were developed separately for males and females. The training samples were based on structural brain measures from 952 males and 1236 female healthy individuals (18–75 years of age) from the ENIGMA Major Depressive Disorder (MDD) group. There is no known participant overlap between the training samples and the participant data used in this work. Briefly, FreeSurfer measures from the left and right hemispheres were combined by calculating the mean ((left + right)/2)) of volumes for subcortical regions and lateral ventricles, and thickness and surface area for cortical regions, resulting in 77 features. The 77 average structural brain measures were used as predictors in a multivariable ridge regression to model chronological age in the healthy training samples (separately for males and females), using the Python-based *sklearn* package [36]. Model performance was originally validated in training samples (through 10-fold cross-validation) and out-of-sample controls. Here, the parameters from the previously trained model(s) were applied to our test samples of healthy controls and SZ patients (and separately for males and females) to obtain brain-based age estimates for each cohort. To assess the model’s generalization performance in the test control samples, we calculated the (1) mean absolute error (MAE) between predicted brain age and chronological age, the (2) Pearson correlation coefficients between predicted brain age and chronological age (*r*), and (3) the proportion of chronological age variance explained by the model (R^2^). For more detailed information on the training samples, model development/validation, and generalisation performance in the current samples, see Supplementary Material and Han et al. [24].

### Statistical analyses

Brain-PAD (predicted brain-based age minus chronological age) was calculated for each participant and used as the outcome variable. While different prediction models were built for males and females, the generated brain-PAD values were pooled across sex for subsequent statistical analyses within each cohort. Each dependent measure of the *i*th individual was modelled as follows:

1 $${{{{{{{\mathrm{brain}}}}}}}} - {{{{{{{\mathrm{PAD}}}}}}}}_{{{{{{{\mathrm{i}}}}}}}} = \, 	{{{{{{{\mathrm{intercept}}}}}}}} + \beta 1\left( {{{{{{{{\mathrm{Dx}}}}}}}}_{{{{{{{\mathrm{i}}}}}}}}} \right) + \beta 2({{{{{{{\mathrm{sex}}}}}}}}_{{{{{{{\mathrm{i}}}}}}}}) + \beta 3\left( {{{{{{{{\mathrm{age}}}}}}}}_{{{{{{{\mathrm{i}}}}}}}}} \right)\\ 	+ \beta 4\left( {{{{{{{{\mathrm{age}}}}}}}}_i^2} \right) + \beta 5\left( {{{{{{{{\mathrm{site}}}}}}}}_{{{{{{{\mathrm{i}}}}}}}}} \right) + \varepsilon _{{{{{{{\mathrm{i}}}}}}}}$$

where Dx represents diagnostic status for SZ. We corrected for the well-documented systematic age bias in brain age prediction (see Supplementary Material for brief explanation of this issue) [37, 38], as well as for potential confounding effects of age and sex in our test samples, by adding age, quadratic age (age^2^), and sex as covariates to our statistical models. We included both linear and quadratic age covariates in the same model as this provided a significantly better model fit to previous data compared with models including a linear age covariate only [24]. In addition, and where applicable, multiple scanning sites/scanners were added as (n-1) dummy variables.

Within SZ patients, we also used linear models to examine associations between brain-PAD and clinical characteristics (CC):

2 $${{{{{{{\mathrm{brain}}}}}}}} - {{{{{{{\mathrm{PAD}}}}}}}}_{{{{{{{\mathrm{i}}}}}}}} = {{{{{{{\mathrm{intercept}}}}}}}} + \beta 1({{{{{{{\mathrm{CC}}}}}}}}_{{{{{{{\mathrm{i}}}}}}}}) + \beta 2\left( {{{{{{{{\mathrm{age}}}}}}}}_{{{{{{{\mathrm{i}}}}}}}}} \right) + \beta 2\left( {{{{{{{{\mathrm{age}}}}}}}}_i^2} \right) + \varepsilon _{{{{{{{\mathrm{i}}}}}}}}$$

where “CC” represents either age of onset, illness duration (time from age-of-onset to time of scanning), SZ symptomatology at study inclusion (including Scale for the Assessment of Negative Symptoms—SANS Global, Scale for the Assessment of Positive Symptoms—SAPS Global, and Positive and Negative Syndrome Scale – PANSS Total), antipsychotic (AP) medication use at time of scanning (typical/atypical/both/none) or chlorpromazine (CPZ) dose equivalents (mg per day). Analyses were also repeated while additionally covarying for handedness (right/left/ambidextrous) or parental socioeconomic status (see Supplementary Material). Cohorts with less than 10 healthy controls and less than 5 participants in a particular predictor or covariate subgroup (e.g., sex, clinical characteristics) were excluded from the analyses (see Supplementary Material for more details).

Cohort-specific results were then meta-analysed using the *rma* function in the *metafor* package [39]. Random (or mixed) effects models were fitted using restricted maximum likelihood estimation and inverse-variance weighting. Statistical tests were two-sided, and results for the effects of nine clinical characteristics among SZ patients were false discovery rate (FDR) corrected (using the Benjamini-Hochberg procedure) and considered statistically significant at *α* < 0.05. In addition, as cohorts differed in age or sex distribution, or, had multiple scanning sites (ASRB, FBIRN, Huilong, MCIC, MPRC, PAFIP) or different MRI scanners, post-hoc meta-regressions were performed to explore between-study heterogeneity in effect size with respect to the number of scanning sites (i.e., single vs. multi-site status), scanner field strength (i.e., 1.5 T vs. 3 T MRI), mean sample age or percentage of females (across cases and controls).

Finally, to better understand the contribution or importance of individual structural brain measures for making brain age predictions, we calculated Pearson’s correlation coefficients between brain-predicted age and each of the 77 FreeSurfer features in each cohort. A weighted average by sample size across cohorts was then calculated for each correlation coefficient and plotted on cortical maps for illustrative purposes only. Correlation analyses were also conducted separately for SZ patients and healthy controls.

## Results

### Sample characteristics

Demographics and clinical characteristics across cohorts can be found in Table 1. Mean age weighted by sample size (range) across SZ patient and healthy control cohorts was 34.22 (18.36–43.66) and 33.82 (22.58–41.41) years, respectively. Patient and control cohorts were on average 67.32% (43.75–100) males and 54.89% (38.46–100) males, respectively. Weighted mean age of onset and duration of illness across patient cohorts were 24.75 (17.55–29.99) and 10.83 (0.62–18.87) years. Mean symptom severity (PANSS total) was 62.41 (33.38–93.12). For cohorts where current antipsychotic medication type information was available, the weighted mean percentage of patients on first-generation (typical), second-generation antipsychotics (atypical), both typical and atypical, or no antipsychotic medication was 10.05%, 67.65%, 14.73% and 7.57%, respectively.

**Table 1:** Participant characteristics for patients and controls across cohorts.

Characteristic	Weighted mean (range)^a^		*K*
	SZ	HC	
Mean % males	67.32% (43.75–100)	54.89% (38.46–100)	26/25
Mean age in (years)	34.22 (18.36–43.66)	33.82 (22.58–41.41)	26/25
Mean age of onset (in years)	24.75 (17.55–29.99)	-	21/-
Mean duration of illness (in years)	10.83 (0.62–18.87)	-	21/-
Mean symptom severity (PANSS total)	62.41 (33.38–93.12)	-	20/-
Mean SANS global	7.94 (3.64–14.06)	-	22/-
Mean SAPS global	6.72 (1.41–12.53)	-	21/-
Antipsychotic medication^b^			*21/-*
Mean % Atypical	67.65% (0.00–93.00)	-	
Mean % Typical	10.05% (0.00–90.24)	-	
Mean % Both atypical & typical	14.73% (0.00–100)	-	
Mean % None	7.57% (0.00–53.62)	-	
Mean CPZ-equivalent dose	414.30 (167.88–1367.94)	-	19
Handedness			*20/19*
Mean % Right	91.15% (81.16–100)	91.05% (81.82–100)	
Mean % Left	6.00% (0.00–14.49)	6.45% (0.00–18.18)	
Mean % Ambidextrous	2.85% (0.00–11.1)	2.49% (0.00–11.67)	

*SZ* patients, *HC* healthy controls, *K* data available for *K* number of cohorts, *SANS* Scale for the Assessment of Negative Symptoms, *SAPS* Scale for the Assessment of Positive Symptoms, *PANSS* Positive and Negative Syndrome Scale, *CPZ* chlorpromazine.

^a^Unless otherwise specified, means are weighted by the number of participants per group (SZ or HC)/cohort. For continuous variables, range indicates the smallest and largest mean value across cohorts. For categorical variables (percentages), range indicates the smallest and largest proportion of participants in each category across cohorts.

^b^Mean percentages are weighted based on the number of cases with recorded antipsychotic type at each cohort.

### Brain age prediction performance

In controls, the weighted average MAE across cohorts was 7.60 (SE = ± 0.40) and 8.45 (SE = ± 0.46) years for males and females, respectively (Supplementary Fig. S2a, b). Within the SZ group, the MAE was 10.14 (SE = ± 0.52) and 9.61 (SE = ± 0.54) years for males and females, respectively (Supplementary Fig. S2c, d). Correlations between chronological age and predicted brain age were moderate to large in controls (males *r* = 0.64, and females *r* = 0.63; both R^2^ = 0.41), and in SZ patients (males *r* = 0.58, and females *r* = 0.62; both R^2^ = 0.33) (Supplementary Fig. S3a–d).

### Brain age differences between SZ and controls

**Fig. 1:**
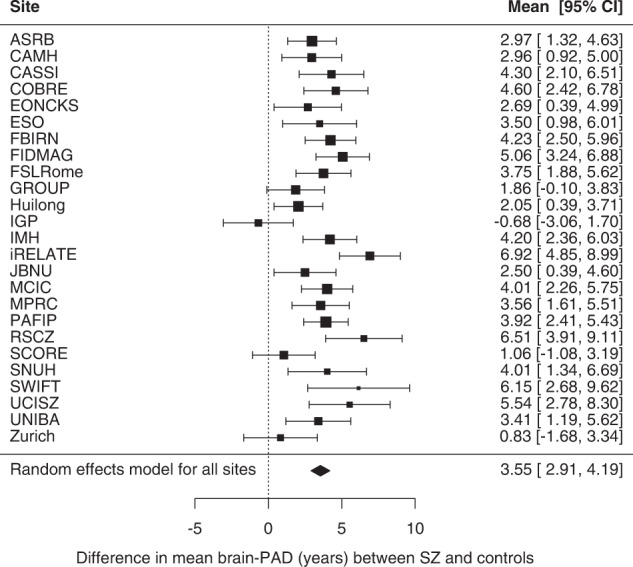
Case-control differences in brain-PAD. Forest plot of differences in mean brain-PAD scores (predicted brain age - chronological age) between patients with schizophrenia (SZ) and controls across (26 −1) 25 cohorts (a total of 2792 cases and 2598 controls; excluding 1 cohort that contributed data for patients only), controlling for sex, age and age^2^ and scanning site. Regression coefficients (in years) are denoted by black boxes. Black lines indicate 95% confidence intervals. The size of the box indicates the weight the cohort received (based on inverse variance weighting). The pooled estimate for all cohorts is represented by a black diamond, with the outer edges of the diamond indicating the confidence interval limits.

Weighted mean brain-PAD scores were +4.39 years (SE = ± 0.84) in the control group and +7.74 years (SE = ± 0.94) in the SZ group. On average, brain-PAD was higher by +3.55 years (95% CI 2.91, 4.19; *p* < 0.0001) in individuals with SZ compared to controls (Cohen’s *d* = 0.48; 95% CI 0.33, 0.63; *p* < 0.0001) adjusted for age, age^2^, sex and scanning site (Fig. 1). Post-hoc sensitivity analysis excluding cohorts in which the model generalised less well (based on MAE > 10.00 or *R*^2^ < 0.1 in healthy controls) returned similar results (see Supplementary Fig. S4). Effect sizes were heterogeneous across individual cohorts (*Q* (24) = 55.15, *p* < 0.0003; *I*^2^ = 57.53%). A significant effect was seen in 22 out of 25 cohorts, with a positive direction of mean effect size observed in all but one cohort. Across cohorts, mean brain-PAD did not differ between single versus multi-site cohorts (QM(1)=0.033, *p* = 0.857), nor between 1.5 T versus 3 T scanners (QM(1) = 0.084; *p* = 0.772) or with respect to mean age (QM(1) = 0.33, *p* = 0.566). There was some evidence for a moderating effect of sex at the cohort level with an attenuated association between SZ and brain-PAD in cohorts with a higher proportion of females (*b* = −0.069, SE = 0.028, QM (1) = 6.271; *p* = 0.012), accounting for some of the residual heterogeneity in the estimated brain-PAD difference between SZ and HC across the 25 cohorts (*R*^2^ = 35.83%; *I*^2^ = 46.68%). We also found a weak linear, yet not significant effect for age on brain-PAD (*b*_age_ = −0.23, 95% CI −0.47, 0.01, *p* = 0.061; *b*_age2_ = −0.00, 95%CI −0.05, 0.05, *p* = 0.998). Additional adjustment for handedness in a smaller pool of 16 cohorts did not meaningfully change our main finding for the effect of SZ (+3.62 years; 95% CI 2.82, 4.42; *p* < 0.0001).

### Brain age and clinical characteristics in SZ

Among SZ patients, we found no statistically significant effects on brain-PAD of clinical characteristics, including age-of-onset, length of illness, symptom severity (PANSS total, SAPS global), antipsychotic use, and CPZ-equivalent dose after adjusting for age and age^2^ (Table 2 and Supplementary Fig. S5a–i). A weak, positive effect for negative symptom severity (SANS global) on Brain-PAD was observed, although it did not reach significance (*b* = 0.18, 95% CI −0.01, 0.38, *P*_FDR_ = 0.62). In addition, no significant effects were found for typical versus atypical and both atypical and typical versus atypical medication groups (Supplementary Table S6). Further adjustment for handedness returned similar results (Supplementary Table S7).

**Table 2:** Clinical characteristics and brain-PAD in individuals with SZ.

Clinical parameter	*N*	*K*	beta	SE	95% CI	*P*_FDR_ value
Age of onset (years)	2053	21	−0.06	0.09	−0.22, 0.11	0.84
Length of illness (years)	2056	21	0.05	0.09	−0.12, 0.22	0.84
PANSS total	1437	20	0.05	0.06	−0.06, 0.17	0.77
SANS global	1911	22	0.18	0.10	−0.01, 0.38	0.62
SAPS global	1892	21	0.14	0.12	−0.09, 0.38	0.70
AP use—atypical vs. unmed	642 (486/156)	7	1.71	1.27	−0.77, 4.19	0.70
AP use—typical vs. unmed	117 (42/72)	3	−0.13	1.00	−2.10, 1.84	0.90
AP use—both vs. unmed	266 (184/82)	4	−0.33	1.08	−2.43, 1.77	0.90
CPZ-equivalent dose	1698	19	0.00	0.01	−0.02, 0.02	0.90

*K* number of cohorts, *N* total number of participants included in each meta-analysis (where applicable, total group size for AP type use/unmedicated is given in brackets), *SE* standard error, *CI* confidence intervals. *P* values are false discovery rate (FDR) adjusted. *SANS* Scale for the Assessment of Negative Symptoms, *SAPS* Scale for the Assessment of Positive Symptoms, *PANSS* Positive and Negative Syndrome Scale, *AP* Antipsychotics, *CPZ* chlorpromazine.

Associations between clinical characteristics and brain-PAD (predicted brain age—chronological age) in SZ. For continuous variables (age of onset, length of illness, PANSS total, SANS/SAPS global and CPZ), the regression coefficient *beta* indicates a mean change in brain-PAD per unit increase in each clinical variable across cohorts. For categorical variables (AP use—typical/atypical/both atypical and typical), beta indicates the mean brain-PAD difference between each treatment group and unmedicated (unmed) individuals. Effects were adjusted for age and age^2^.

### Correlations between brain imaging features and brain age

**Fig. 2:**
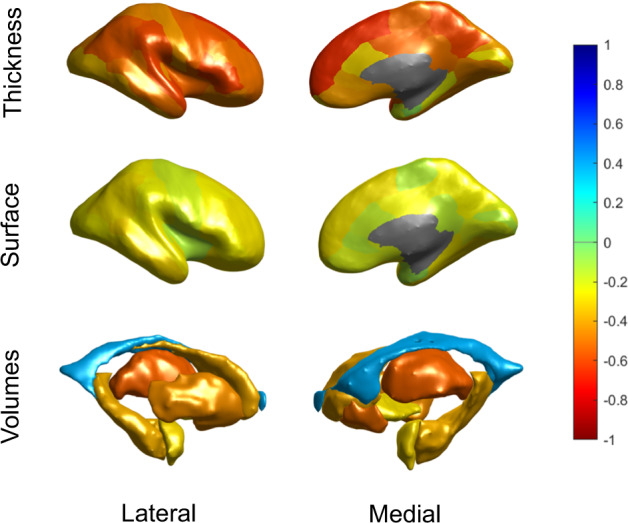
Correlation coefficients of predicted brain age and FreeSurfer features across control and schizophrenia (SZ) groups. Bivariate correlations are shown to provide an indication of the relative contribution of features in brain age prediction. The figure shows Pearson correlations between predicted brain age and cortical thickness features (top row), cortical surface areas (middle row) and subcortical volumes (bottom row), from both the lateral (left) and medial (right) view. Features were averaged across the left and right hemispheres. The negative correlation with ICV was excluded from this figure for display purposes.

All imaging features, except mean lateral ventricle volume, were negatively correlated with predicted brain age (Fig. 2); thickness features correlated more strongly with brain age (mean Pearson *r* [SD]: − 0.46 [0.13]), especially in medial frontal and temporo-parietal regions, than subcortical volumes (−0.32 [0.30]) or surface area features (−0.22 [0.06]). We also visualized these associations separately for controls and SZ patients with similar results, suggesting comparable structure coefficients in both groups (for more details see Supplementary Material).

## Discussion

We assessed brain ageing in 2803 individuals with SZ and 2598 healthy controls using a novel brain age algorithm based on FreeSurfer ROIs. Results indicate that, at a group level, patients with SZ show a greater discrepancy between their brain-predicted age and chronological age compared to healthy individuals (+3.55 years), with a moderate increase in brain-PAD (Cohen’s = 0.48). The greater brain-PAD in the SZ group was not driven by any of the specific clinical characteristics assessed here (age of onset, length of illness, symptom severity, and antipsychotic use and dose). This study has two major strengths. Firstly, through a prospective meta-analytic approach within the ENIGMA consortium, we were able to assess brain age differences between SZ patients and healthy controls using standardised analysis methods across multiple independent cohorts worldwide, providing a generalised mean effect size. Second, the overall large sample size and harmonisation of data across cohorts allowed for a more reliable assessment of the relationship between clinical variables and brain-PAD among SZ patients.

The mean brain-PAD difference between patients and controls was +3.55 years (Cohen’s *d* = 0.48) in our study. Overall, this finding is aligned with previously reported brain-PAD scores in SZ patients vs. healthy controls (range: +2.6–7.8 years) [26–33]. Schnack et al. [29] and a recent mega-analysis by Kaufmann et al. [32] found similar effect sizes (+3.4 years and Cohen’s *d* = 0.51, respectively) in largely non-overlapping/independent samples from this current study. On the other hand, our brain-PAD difference is smaller relative to that reported in earlier work by Koutsouleris et al. [27] and Shahab et al. [30] showing respectively +5.5 to +7.8 years of brain age in smaller samples of SZ patients. Several methodological differences may explain the variability in magnitude of brain age effects in SZ across studies, including the type of neuroimaging features (e.g., voxel-wise vs. ROI-based morphometric data; and/or single vs. multiple imaging modalities) [40], the machine learning algorithm used for brain age estimation [41], the size of training and test data samples, and differences in patient characteristics.

Relative to healthy controls, brain-PAD scores in SZ suggest more advanced brain ageing than in MDD (+1.12 years) [42] and bipolar disorder (BD; +1.93 years) [42], that may reflect more pronounced structural brain abnormalities in SZ [24]. This aligns with previous reports from the ENIGMA consortium, showing largest effect sizes of cortical and subcortical gray matter alterations in SZ (highest Cohen’s *d* effect size = 0.53) [16, 17], followed by BD (highest Cohen’s *d* = 0.32) [43, 44] and MDD (highest Cohen’s *d* = 0.14) [45, 46]. Hence, sensitivity of brain-PAD to SZ at the group level appears to be quantitively similar to that of leading cortical thickness and subcortical volume measures. A further key advantage of the “brain age” paradigm is that it captures multivariate age-related structural brain patterns into one (or more) composite measure(s), thereby simplifying analyses and aids interpretation with respect to normative patterns of brain ageing.

Consistent with previous reports [27, 31], we did not observe significant associations between brain-PAD and age of onset, length of illness, and antipsychotic treatment or dose among SZ patients. This suggests that a greater brain-PAD in SZ may not be primarily driven by disease progression or treatment-related effects on brain structure that have been reported elsewhere [12, 14, 18, 47, 48]. This is in keeping with previous studies showing a greater brain-PAD already present in first-episode SZ and first-episode psychosis patients [26, 49]. Using a longitudinal design, Schnack et al. investigated brain age acceleration (i.e., annual rate of change in brain-PAD) over the duration of illness in SZ (*N* = 341; mean follow up period: 3.48 years). Brain-PAD started increasing by about 2.5 years (per year) just after illness onset, though this acceleration rate slowed down to a normal rate over the first 5 years of illness [29]. Lastly, in contrast to previous findings in SZ [27] and first-episode psychosis [49] we did not find strong evidence for a positive association between negative symptom severity and brain-PAD. An explanation for this could be that negative symptoms are more specifically linked to brain age differences at the regional level (i.e., temporal or parietal brain-PAD) than at the global level (i.e., “whole-brain” brain-PAD), as reported previously [32].

The biological mechanisms underlying advanced brain ageing in SZ remain elusive. These may involve interrelated biochemical abnormalities that accompany both schizophrenia and brain ageing, including increased inflammation and oxidative stress [10, 50]. Elevated levels of inflammatory markers (e.g., pro-inflammatory cytokines in blood and central nervous system) have been observed by multiple studies in individuals with schizophrenia [11, 51]. Moreover, there has been evidence for peripheral inflammation markers being associated with structural brain abnormalities observed in schizophrenia and related outcomes (e.g., first episode psychosis), including but not limited to abnormal cortical thickness of the bilateral Broca’s area and temporal gyrus [52, 53], as well as with greater brain-PAD scores [54]. Abnormal levels of multiple oxidative stress markers have also been observed in SZ, both peripherally and in brain tissue [11, 55]. Oxidative stress and inflammation may reciprocally induce one another via a positive feedback loop in SZ, resulting in cellular damage [56].

Several methodological issues require further consideration. First, while a brain-PAD score (that is not equal to zero) is conceptually a prediction error that could reflect physiological deviations from normal ageing trajectories, it could be partly attributed to lack of model accuracy due to noise or unwanted variation [32, 57, 58]. Potential sources of unwanted variation include the use of multiple scanners and/or image acquisition protocols across (or within) participating cohorts that may affect the overall generalization performance of the brain age model applied here. To overcome this, in the primary analysis we included cohorts that had data on both cases and healthy controls collected in a similar, if not identical, manner (i.e., same site/scanner and/or image acquisition protocol) and have adjusted for multiple scanners where applicable. Nevertheless, while our model fit is lower than some previous studies, this would only increase noise, not a bias towards finding an effect of SZ on brain-PAD. Second, although our meta-analytic approach allowed us to combine information across multiple cohorts, the summary-level data reported here does not adequately capture the considerable inter-individual variability in brain-PAD among SZ patients, as has been documented elsewhere [32]. As some individuals with SZ are not characterised by a greater brain-PAD, it would be important to further investigate both clinical as well as biological, lifestyle and technical confounding factors that are linked to SZ and/or brain-PAD (e.g., inflammation, smoking, body mass index, imaging parameters) potentially accounting for inter-individual variability. Given that greater brain-PAD has been associated with poorer health outcomes, such as an increased mortality risk [23], understanding the extent to which various factors may contribute to brain ageing in SZ could help prioritize targets for interventions aiming to halt (or reverse) advanced brain ageing. Additionally, future studies should direct their efforts towards better characterization of region-specific brain patterns that could explain individual variation as well as differences in (global) brain-PAD within and between groups [59, 60]. Third, although the sample size of our main analysis (SZ versus controls) was very large for a neuroimaging study, the size of patient groups categorised by status of antipsychotic use was relatively small (particularly that of unmedicated individuals with SZ) and cohort differences include the use of different assessments or processes to ascertain medication use and dose. This may have precluded detection of some associations. Lastly, given the cross-sectional design of the current study, we were not able to assess brain age acceleration more directly and how that may be related to clinical characteristics. Longitudinal large-scale studies are better suited for examining brain ageing per se [61] and for evaluating the clinical relevance of brain-PAD in SZ.

In conclusion, we found evidence of advanced brain ageing in SZ patients compared to healthy controls, which does not seem to be driven by the effects of medication or other clinical characteristics. Deviations from normative brain ageing trajectories in SZ may at least in part reflect increased risk of premature mortality and age-related chronic diseases commonly seen in SZ. Future longitudinal studies with more in-depth clinical characterization—including information on mental and somatic health outcomes—will be needed to elucidate whether a brain age predictor such as brain-PAD can provide a clinically useful biomarker to inform early prevention or intervention strategies in SZ.

## Supplementary information

Supplementary Materials

Supplementary Tables and Figures

## Data Availability

The R code used to perform the individual-level analyses described above is openly available on GitHub: https://github.com/ConstantinosConst/ENIGMA-SZ-BrainAge. Further information can be requested from the corresponding author.
