# Supplementary material for: Brain ageing in schizophrenia: evidence from 26 international cohorts via the ENIGMA Schizophrenia consortium
Source: Mol Psychiatry. 2022 Dec 9;28(3):1201–9. doi: 10.1038/s41380-022-01897-w (PMC10005935; doi:10.1038/s41380-022-01897-w)
Supplement: Supplementary file 1 — Supplementary Materials [file 41380_2022_1897_MOESM1_ESM.docx]

**Supplementary Material**

**Image exclusion criteria**

As described in Han *et al.* [1], a neuroimaging expert at each scanning site inspected each image segmentation by overlaying the segmentation label of each structure on the T1-weighted brain scan. Additionally, study-wide statistics were collected (means and standard deviations) as well as histogram plots to identify non-normally distributed data and major outliers. Samples were marked if its FreeSurfer feature was >2.698 standard deviations away from the global mean. If a sample was marked as a statistical outlier, the individual site was asked to re-inspect the subject’s segmentation to verify that it was properly segmented. If a sample was a statistical outlier, yet properly segmented, it was kept in the dataset. Otherwise, the sample was removed. Out of 26 cohorts included in the current study, 10 had information available on the number and/or reasons for exclusion (see Supplementary Table S4 for more details). In the full sample of included and excluded participants (N=5475; K=26), N=74 (1.35%) participants were excluded due to image QC. Of those 74 excluded participants, 29 (39%) were controls. Where information was available, excluded participants were more likely to be male.

**Quality checking and sample exclusion criteria**

For each of the participating cohorts, we excluded those participants below 18 years and above 75 years old (if any) to focus on adult patients and controls, and to match the age range of the training dataset previously used to develop the ENIGMA brain age prediction model (see Han *et al*. [1] for further details on the rationale for this age range). There were similarities and differences between the training samples used in Han et al., and the testing samples of the current study. Compared to Han et al., weighted mean age across the current cohorts was slightly younger and more samples were acquired using a 3T scanner. However, across both studies, most cohorts were from Europe and most data were acquired using a sagittal slice orientation and FreeSurfer version 5.3*.* We checked individual FreeSurfer features for missing values and excluded participant samples with >10% missing data, suggestive of poor reliability. In addition, following calculation of brain-PAD for each participant, we intended to winsorize extreme brain-PAD outliers - here defined as data points outside the 5*interquartile range (IQR) within each cohort. However, we identified no outliers outside the 5*IQR at each cohort.

Cohorts with less than 5 participants per group with respect to diagnosis (SZ/HC) or sex (males/females) were excluded from subsequent analyses. As a result of this criterion, one cohort was excluded from the primary case-control analysis due to a HC sample size of less than 5, but SZ data from this site (n=11) contributed to additional analyses (i.e., within the SZ group). Note that for any given clinical characteristic assessed (age of onset, length of illness, symptom severity scores, and antipsychotic use and dose), some of the participating cohorts had no data available (see Supplementary Tables S1 and S2 for more details) and thus were automatically excluded from additional analyses. In addition, cohorts with available data on less than 5 SZ patients per clinical characteristic (or subgroups for antipsychotic use) were excluded. This resulted in 6 cohorts being excluded from additional analyses on the effects of antipsychotic use.

**Data harmonization for clinical characteristics**

Symptom severity was assessed using the Scale for the Assessment of Negative Symptoms (SANS) [2], the Scale for the Assessment of Positive Symptoms (SAPS) [3], and the Positive and Negative Syndrome Scale (PANSS) [4]. To harmonise symptom severity scores, we decided to convert all positive (i.e., PANSS-Positive and total SAPS composite scores) and negative (i.e., PANSS-Negative and total SANS composite scores) to Global SAPS and Global SANS (summary) scores, respectively. These conversions were made based on recommendations by Andersen [2, 3] and using the algorithms published in van Erp *et al*. [5]. In addition, data on medication dose for typical and atypical antipsychotics were harmonised based on chlorpromazine (CPZ) dose equivalents, as described by Woods ([www.scottwilliamwoods.com/files/%0DEquivtext.doc](http://www.scottwilliamwoods.com/files/%0DEquivtext.doc)).

**Model validation**

As described in Han *et al*. [1], tenfold cross-validation was performed in the training sample of healthy controls (from the ENIGMA MDD working group) to assess model performance. To quantify model performance, we calculated the (1) mean absolute error (MAE) between predicted brain age and chronological age, (2) Pearson correlation coefficients between predicted brain age and chronological age(r), and (3) the proportion of the variance explained by the model (R^2^). The accuracy of the model was further validated using a hold-out dataset of healthy controls from the same scanning sites as the training dataset (males, N = 927; females, N = 1199). To evaluate generalizability of the model, independent control test samples (acquired on completely independent scanning sites) from the ENIGMA-BD working group were used. Training model parameters were applied on these independent subjects (males, N = 610; females, N = 720) from the ENIGMA BD working group.

Within the training set of controls, under cross-validation, the structural brain measures predicted chronological age with a MAE of 6.32 (SD 5.06) years in males and 6.59 (5.14) years in females. Correlation between chronological age and predicted brain age in the cross-validation training sample was r = 0.85, p < 0.001 for both males and females (both R^2^ = 0.72). Model performance in the hold-out dataset (males MAE = 6.50 [SD 4.91]; r = 0.85, p < 0.001; R^2^ = 0.72 and females MAE = 6.84 [5.32]; r = 0.72, p < 0.001; R^2^ = 0.69) was comparable to that of the cross-validation training sample. When applying the model parameters to the independent healthy control samples of the ENIGMA BD working group the generalization worked fairly well (MAE = 7.49 [SD 5.89]; r = 0.71, p < 0.001; R^2^ = 0.45 for males and MAE = 7.26 [5.63]; r = 0.72, p < 0.001; R^2^ = 0.48, for females) (for more details see Han *et al*. [1]).

In terms of the model’s performance in our independent test control samples, Supplementary Table S5 provides a summary of the model performance metrics for the hold-out (test) control samples from Han et al. and the overall current control sample (for males and females, separately). Overall, the model performed well in the current control sample. However, the brain age model was applied to each cohort separately, and model performance metrics varied considerably between cohorts (see Supplementary Figures S2a-b and S3a-b). Factors that could explain between-cohort differences in model performance metrics include differences (or similarities) in age range/distribution, scanner, and exclusion/inclusion criteria for controls between the training samples from Han et al and each testing sample in the current study. See de Lange et al (2022) for a thorough discussion on evaluating brain age model performance and its limitations [9]*.*

**Correcting for the systemic age bias in brain-age prediction in subsequent analyses**

“Regression dilution” is a well-known phenomenon in any brain age prediction framework, which often leads to young people being systematically predicted to be of older age (than their chronological one) and older people to be systematically predicted younger than their chronological age [6–9]. To account for this potential bias, we have included chronological age as a covariate in subsequent analyses, as proposed elsewhere [6]. This removes all linear age effects on our outcome variable (brain-PAD). However, one cannot assume that the effect of ageing on imaging measures is perfectly linear across the lifespan [7, 10, 11]. Therefore, to control for potential non-linear age effects on brain age estimation and brain-PAD, we also included quadratic age (age^2^) in our subsequent analyses, as suggested elsewhere [7].

**Additional adjustment for socioeconomic status**

As an extension to our primary analysis, we explored parental socioeconomic status (SES) as a potential confounder of the association between SZ and brain-PAD. We have chosen to focus on parental SES rather than own (participant) SES, as the socioeconomic status of the parent(s) is less likely to be causally affected by the disorder. Parental SES was obtained from a subset of eight cohorts (CAMH, COBRE, FBIRN, MCIC, PAFIP, SNUH, UCISZ, UNIBA) and was assessed based on education level, occupation and/or income of the parent(s), mainly derived from the Hollingshead four-factor index of SES [12]. Among these eight samples, parental SES was significantly higher/lower/non-differential (at an uncorrected alpha = 0.05) in patients compared to controls in 0/3/5 samples, providing mixed results for an association between parental SES and SZ. Parental SES was/was not significantly correlated with brain-PAD in 1/7 samples. We then ran a multiple linear regression additionally adjusting for SES in this subset of 8 cohorts using the following model:

Brain-PAD_i_ = intercept + β1(Dx_i_) + β2(sex_i_) + β3(age_i_) + β4(age^2^_i_) + β5(site_i_) + β6 (**parental SES_i_**) + ε_i_

(number of scanning sites were added as (n-1) dummy variables)

The results of our primary meta-analysis with and without adjustment for parental SES are provided in Supplementary Figure S6. The mean difference in overall effect point estimate of SZ between the unadjusted and unadjusted model for parental SES is -0.15. The results of this exploratory analysis do not indicate that the observed relationship between SZ and brain-PAD is substantially confounded by parental SES.

**Feature importance: correlations between brain imaging features and brain age**

All imaging features, except the mean lateral ventricle volume, were negatively correlated with predicted brain age (and are visualised in Figure 2 in the main manuscript), although thickness features correlated more strongly with brain age (mean Pearson r [SD]: −0.46 [0.13]) than subcortical volumes (−0.32 [0.30]) or surface area features (−0.22 [0.06]). We also visualized these associations separately for controls and SZ patients with similar results, suggesting comparable structure coefficients in both groups (see Supplementary Figure S7). With regards to spatial patterns of brain ageing, our correlation analysis of individual FreeSurfer measures and brain-predicted age does not allow for a straightforward interpretation of the importance of specific brain regions contributing to the underlying ageing pattern (i.e., for the same reason we did not include a spatial weight map of our brain age model, as the weights were obtained from a multivariable model). Nevertheless, our results indicate that the ENIGMA brain age model used here relied most heavily on cortical thickness features for making predictions - consistent with the results of its first report by Han *et al*. [1]. The widespread and relatively strong negative correlations between cortical thickness and brain age (as observed here) are in alignment with the general pattern of greater cortical thinning with advancing age reported in the existing literature [13].

**References**

1. Han LKM, Dinga R, Hahn T, Ching CRK, Eyler LT, Aftanas L, et al. Brain aging in major depressive disorder: results from the ENIGMA major depressive disorder working group. Mol Psychiatry. 2020:1–16.

2. Andreasen NC. Scale for the assessment of negative symptoms (SANS). Iowa City Univ Iowa. 1984. 1984.

3. Andreasen NC. Scale for the assessment of positive symptoms (SAPS). Iowa City Univ Iowa. 1984. 1984.

4. Kay SR, Fiszbein A, Opler LA. The positive and negative syndrome scale (PANSS) for schizophrenia. Schizophr Bull. 1987;13:261–276.

5. van Erp TGM, Preda A, Nguyen D, Faziola L, Turner J, Bustillo J, et al. Converting positive and negative symptom scores between PANSS and SAPS/SANS. Schizophr Res. 2014;152:289–294.

6. Le TT, Kuplicki RT, McKinney BA, Yeh H-W, Thompson WK, Paulus MP. A Nonlinear Simulation Framework Supports Adjusting for Age When Analyzing BrainAGE. Front Aging Neurosci. 2018;10:317.

7. Smith SM, Vidaurre D, Alfaro-Almagro F, Nichols TE, Miller KL. Estimation of brain age delta from brain imaging. Neuroimage. 2019;200:528–539.

8. Liang H, Zhang F, Niu X. Investigating systematic bias in brain age estimation with application to post-traumatic stress disorders. Hum Brain Mapp. 2019;40:3143–3152.

9. de Lange AMG, Anatürk M, Rokicki J, Han LKM, Franke K, Alnæs D, et al. Mind the gap: Performance metric evaluation in brain-age prediction. Hum Brain Mapp. 2022;43:3113–3129.

10. Fjell AM, Westlye LT, Grydeland H, Amlien I, Espeseth T, Reinvang I, et al. Critical ages in the life-course of the adult brain: nonlinear subcortical aging. Neurobiol Aging. 2013;34:2239.

11. Wierenga LM, Langen M, Oranje B, Durston S. Unique developmental trajectories of cortical thickness and surface area. Neuroimage. 2014;87:120–126.

12. Hollingshead, AA. Four-factor index of social status. 1975; Unpublished manuscript, Yale University, New Haven, CT.

13. Frangou S, Modabbernia A, Williams SCR, Papachristou E, Doucet GE, Agartz I, et al. Cortical thickness across the lifespan: Data from 17,075 healthy individuals aged 3–90 years. Hum Brain Mapp. 2021;31:14.
